# Proceedings of the American Pharmaceutical Association, at the Eighth Annual Meeting, Boston, Mass., Sept., 1859

**Published:** 1860-05

**Authors:** 


*Proceedings of the American Pharmaceutical Association, at the Eighth Annual Meeting, Boston, Mass., September*, 1859. Boston: Geo. C. Rand & Avery. 1859. Pp. 416.

The proceedings of this active, energetic body have been on our table for some time, and should have received notice before this, but other books and other subjects have required our attention, so that the volume containing them has been laid aside until the present. After several days spent in deliberating over topics of interest to Pharmacy, while enjoying the hospitality of their Boston brethren, the American Pharmaceutists adjourned, to meet in New York Sept. 11, 1860. Their Proceedings, as contained in the volume before us, are specially valuable on account of the Reports of Committees being given in full, so as to show how active and business-like are the operations of the Association. A glance at the nature of some of these reports will show what the Association is furnishing at its annual meetings.

The *Report on Progress of Pharmacy* is a well-prepared abstract of all the discoveries and improvements in Pharmacy, published during the year ending with the period of the meeting. To those who have not ready access to many journals, (and what physician or apothecary has?) this report gives a bird's-eye view of all that is interesting or attractive, collected in such a condensed form that but little time is required to master it in full. On account of this abstract alone the volume is worth the price charged for it. But while this report is of practical use, we cannot say quite so much for that of the Committee on Weights and Measures. Much learning and laborious research have been employed in its preparation, but it seems to us as much out of place in this volume, as a treatise on *Quaternions* would be in the midst of an ordinary school Arithmetic. True it is, that there should be *international* uniformity, and that a national congress called for this purpose would pave the way for such a result, but we cannot even dream of the entire destruction of decimal arithmetic, of the abolition of the present system of arithmetical computation by tens. The author of this report, however, sets forth the objections to this system in computation, as fourths, eighths, &c., can only be obtained by the aid of fractions. He considers that an octonary series would obviate all this, and make our arithmetical labors more simple and intelligible. On an octonary basis, he would construct all our tables of weights and measures. This would require *new* phraseology, a specimen of which is as follows:

| Un. | Du. | The. | Fo. | Pa. | Se. | Ki. | Unty. |
|---|---|---|---|---|---|---|---|
| 1 | 2 | 3 | 4 | 5 | 6 | 7 | 8 |

The octades are named by the euphonious titles,

| Duty. | Thety. | Foty. | Paty. | Sety. | Kity. | Under. |
|---|---|---|---|---|---|---|
| 16 | 24 | 32 | 40 | 48 | 56 | 64 |

We doubt very much whether all the advantages of such a system would induce the world to commence learning new Numeration and Multiplication tables.

*The Report on the Revision of the Pharmacopœia* contains the sugges tions of such practical men as Parrish, Grahame, and Carney, as to the necessary changes which should be made by the decennial convention. *The Report on Home Adulterations* satisfies us of the increasing insensibility to truth and honor, in the case of some dealers in drugs and in articles required for table consumption. When shall the public be protected from rascality by proper legislation ?

There are also a number of valuable *special* reports, made by members of the association, which are generally *real* contributions to knowledge. That on Silphium laciniatum, *rosin weed*, seems wonderfully *out of place* among the other reports The object is to show the character of the silphium, and to answer the questions, " Can it be substituted for mastic ? To what extent may it be collected as an article of commerce ?" The author seems to have obtained about an ounce. He pronounces it preferable in taste to mastic; but speaks of its properties in allaying irritation of the lungs and checking coughing, not from having tried it, but from having tried mastic. To confirm his own " reasoning from analogy," he quotes the case of a young lady " who was troubled with very weak lungs, so much, in fact, that the difficulty was a source of anxiety to her friends, and she herself was fearful that she would be compelled to relinquish her employment as a teacher; for often, at the close of a day, she was scarcely able to articulate above a whisper." Fortunately, she lived " where the rosin weed was plentiful," and she "*got to chewing the gum.*" She eventually became perfectly cured, and has to-day as strong and healthy a pair of lungs as could be desired." After this *scientific* statement, who can doubt the value of rosin weed ? Who will hesitate to believe, that " were the virtue of this unpretending weed generally known, many valuable lives might be saved that now yield to the insidious and persevering *demolutions* of that destroyer, consumption ?" " *Werry true*, Sammy," as old Mr. Weller would say. We were about quoting some sentences from the same report, describing a fire on the prairie, consuming the rosin weed, but—we forbear. If our friend of the *Knickerbocker* wants something graphic on this subject, we refer him to the report itself. s.

